# Shared Decision-Making and Information Needs among People with Generalized Anxiety Disorder

**DOI:** 10.3390/ejihpe11020031

**Published:** 2021-05-21

**Authors:** Vanesa Ramos-García, Amado Rivero-Santana, Andrea Duarte-Díaz, Lilisbeth Perestelo-Pérez, Wenceslao Peñate-Castro, Yolanda Álvarez-Pérez, Ana Isabel González-González, Pedro Serrano-Aguilar

**Affiliations:** 1 Canary Islands Health Research Institute Foundation, 35019 Tenerife, Spain; vanesa.ramosgarcia@sescs.es (V.R.-G.); amado.riverosantana@sescs.es (A.R.-S.); andrea.duartediaz@sescs.es (A.D.-D.); yolanda.alvarezperez@sescs.es (Y.Á.-P.); 2 Faculty of Health Sciences, University of La Laguna, Campus de Guajara, 38200 Tenerife, Spain; 3 Research Network on Health Services in Chronic Diseases (REDISSEC), 48010 Tenerife, Spain; lilisbeth.peresteloperez@sescs.es (L.P.-P.); pseragu@gobiernodecanarias.org (P.S.-A.); 4 Evaluation Unit (SESCS), Canary Islands Health Service (SCS), 38109 Tenerife, Spain; 5 Institute of General Practice, Goethe University, D-60590 Frankfurt, Germany; aisabel.gonzalezg@salud.madrid.org; 6 General Subdirectorate for Health Research and Documentation, Community of Madrid Health Service, 28013 Madrid, Spain

**Keywords:** generalized anxiety disorder, information needs, patient decision aids, shared decision making, internet

## Abstract

Shared decision making (SDM) aims to involve patients in the decisions about their care, considering their preferences, values and concerns about the different treatment options. However, research shows that people with mental health problems have considerable unmet information needs about their condition. This community-based cross-sectional study explores the SDM process and information needs among people with Generalized Anxiety Disorder (GAD), as an initial step in the design and development of a Patient Decision Aid for this population. Seventy participants completed an online survey with the Control Preference Scale, and questions about the perceived difficulty of past treatment decisions and the use of the Internet for searching for GAD-related information. Most participants preferred an active (42.9%) or collaborative role (41.4%) in the SDM process, and 53% did not perceive their preferred role. Information provided by healthcare professionals was considered insufficient by 28% of the sample, and over 30% reported using the Internet to look for GAD-related information at least once a week or more. The most relevant GAD-related information needs were general information (71.4%), information on self-help groups (65.7%), recommendations on how to face this disorder (61.4%) and information on treatment options (50%). Exploratory analyses showed that patients who perceived an active participation were more likely to search for information frequently (*p* = 0.038), and those who felt more involved than desired tended to search for more themes (*p* = 0.049). In summary, the study showed that a considerable percentage of GAD patients have unmet needs related to decision-making participation and information.

## 1. Introduction

The World Health Organization (WHO) reported in 2017 more than 260 million people worldwide living with Anxiety Disorder (AD) [1]. In Spain, around 2 million people (4.1% of the population) suffer from these disorders [2]. Among AD, Generalized Anxiety Disorder (GAD) is one of the most common, with a twofold prevalence in women compared to men [3].

GAD involves excessive and uncontrollable fear and worries about a wide range of situations and matters, and a constant feeling of being overwhelmed that interferes with daily activities. Physical symptoms such as restlessness, feeling on edge or easily fatigued, difficulty concentrating, muscle tension or sleep problems may be also associated [4]. GAD symptoms generate an important functional and cognitive impairment and a decrease in health-related quality of life [4]. Evidence-based treatments for GAD include psychological (e.g., cognitive behavioral therapy and mindfulness) and pharmacological interventions (selective inhibitors of serotonin reuptake (SSRIs)) [5,6,7].

Person-centered care is currently considered the gold standard of health care [8], and shared decision-making (SDM) between patients and health care providers is a core component of this model. SDM aims to establish a collaborative dialogue between the two agents, in which patients’ values, preferences and concerns about the different available treatment options are taken into account and incorporated into the decision-making process [9]. SDM aims to reach a *good quality decision* about treatment, defined as a decision which is: (1) based on scientific evidence and (2) concordant with patient’s preferences and values [10]. Interventions based on SDM, such as Patient Decision Aids (PtDAs), have shown to improve patients’ knowledge about available treatments and its benefits/risks, decisional conflict, concordance between patients’ preferences and choices, satisfaction and other variables related to the decisional process [11]. Different studies have observed that mental health patients, including those with AD, are willing to participate and become involved in the decisions about their care, a desire that is often not satisfied [12,13,14]. Unfortunately, the number of interventions designed to promote SDM in the field of mental health disorders, and specifically GAD, are still scarce [15,16].

A necessary requirement for the implementation of a successful SDM process is to be aware of patients’ information needs, in order to provide evidence-based information by means of PtDAs or training of professionals [17]. Clinical Practice Guidelines (CPG) have also begun to incorporate people’s values and preferences in order to embrace a more person-centered approach in the decision-making process [18]. However, research on people’s information needs in the field of mental disorders is still lacking. A systematic review published in 2015 [19] included 12 studies assessing information needs among people with mental health problems, but only one [20] considered AD. Results showed that patients with depression or schizophrenia perceive a high need for information in a wide range of topics concerning medication treatment, treatment setting, general treatment issues, non-pharmacological treatment, work, living conditions and lifestyle. The same year, Liebherz et al. [21] published the results of a survey of AD patients where they asked about online health information needs and preferences in decision-making. The most relevant needs were general information, information about treatment options and tips on dealing with the disease. The majority of patients preferred to share decisions with their clinicians, but they reported to be less involved than they would have liked to be. Similarly, the qualitative study by Kivelitz et al. [22] found that patients with mental disorders wanted to be involved in the SDM process and to be informed with useful PtDAs about treatment options.

This study is part of the initial phase of the research project: “*Shared decision making for GAD patients in primary care” (Clinical Trials Registry number: NCT04364958)*, whose main objective is to develop and evaluate the effectiveness of an online PtDA for people with GAD. In this initial phase of the project, we aimed to explore the SDM process and information needs among people with a current or past diagnosis of GAD. Specifically, the objectives of this study are: (1) to assess patients’ preferences and perceptions of being involved in treatment decisions, and their sociodemographic predictors; (2) to assess patients’ information needs about GAD; (3) to explore the associations between preferences/perceptions of being involved (and their matching) with the perceived difficulty of past decisions about GAD treatment, the frequency of searching GAD-related information on the Internet and information needs.

## 2. Material and Methods

### 2.1. Ethics Approval and Consent to Participate

The Scientific and Ethics Committee of the University Hospital Nuestra Señora de la Candelaria (Tenerife, Spain) approved the study (file number: CHUNSC_2019_58). The study has been performed in accordance with Good Clinical Practice standards, applicable local regulatory requirements and the recommendations of the Declaration of Helsinki (2013 version).

### 2.2. Study Design

We conducted an online community-based cross-sectional study, using a purpose-built survey addressing people with GAD in order to assess their information and decision-support needs and to know how they use the Internet to satisfy them.

### 2.3. Participants and Setting

The study was conducted with the Spanish population. Inclusion criteria were adults (18 years of age or older) with a current or past diagnosis of GAD, based on the Diagnostic and Statistical Manual of Mental Disorders (DSM) [3] or the International Statistical Classification of Diseases (ICD) [23]. We excluded people with other mental health diagnoses and patients with AD different from GAD (i.e., separation anxiety disorder, specific phobia, social anxiety disorder, panic disorder, agoraphobia, substance/medication-induced anxiety disorder and anxiety disorder due to another medical condition). Prior to acceptance to participate, eligible patients received study information and provided written consent.

### 2.4. Recruitment

In order to identify eligible participants, we used purposive sampling to select mental health professionals and primary care clinicians with expertise in the management of GAD. A member of the research team contacted them, directly or through professional associations, to provide information about the study aims and invite them to participate by selecting those people meeting the inclusion criteria. They were also asked to share with participants the project information sheet and the hyperlink providing access to the online survey, including the informed consent on the first page. We selected participants with either a current or past diagnosis of GAD.

To reduce a possible potential selection bias by the health professionals, the Amtaes Patient Association published a recruitment advertisement on their website. The Amtaes Patient Association requests a diagnosis from a clinician in order for the patient with GAD to become part of this association. Eligible patients with GAD could apply and would subsequently receive a hyperlink to the online survey if they met the selection criteria.

### 2.5. Survey Development and Data Collection

A survey was created using SurveyMonkey^®^ and sent by email to all those who agreed to participate in the study. An initial invitation email to fill in the survey was sent in early April 2020, and a reminder was sent four weeks later. The survey was open for four months, from 15th April to 14th July. The questionnaire assessed sociodemographic variables and clinical data (see Appendix A).

In addition, to examine the four following dimensions of interest, we included specific instruments:

#### 2.5.1. Role in Decision-Making

We used two items adapted from the Control Preference Scale (CPS) [16] to assess preferred and perceived roles, respectively. Each one had five statements that ranged from completely active to completely passive, with a shared role in the midpoint. Scores were recoded into three categories (active, collaborative, passive), and a new variable was created (matching) to reflect the concordance between preferred and perceived roles (three categories: less involvement than desired, matched, more involvement than desired).

#### 2.5.2. Treatment Decisions

This questionnaire included eleven decisions in four categories: wait-and-see (1), pharmacological (5), psychological (4) and combined (1) treatments, developed ad hoc for this study. For each decision, participants were asked if they had to make it in the past and, if so, how difficult it was for them, rated on a 4-point scale, from 0 (very easy) to 3 (very difficult) [21]. For the pharmacological and psychological decisions, respectively, we calculated for each participant the mean difficulty of the decisions they had taken.

#### 2.5.3. Frequency of Information Search

Participants were asked how often they look for GAD-related information on the Internet. Response options were: *“daily”*, *“2–3 times a week”*, *“once a week”*, *“once a month”*, *“less than once a month”* or *“never”*.

#### 2.5.4. Information Needs

Two items adapted from the survey of Liebherz et al. [21] were used to assess what information participants had looked for on the Internet and for what reasons, respectively. Response options were yes/no (an open-ended question to report other themes/reasons was also included). Reasons included: (1) interest in learning about GAD, (2) doubts about GAD, (3) information given by the health care provider was not enough, (4) it was difficult to understand, (5) they disagreed with the information, (6) they were told to read about GAD by their healthcare professional, (7) they were looking for a healthcare professional. Themes included: (1) general information about GAD, (2) diagnosis, (3) treatment options, (4) risks and benefits of each treatment, (5) where they could receive treatment (setting), (6) support groups, (7) information for family members and (8) recommendations for coping with GAD. The total number of themes was calculated for each individual.

## 3. Data Analysis

We performed descriptive analyses for all the included variables (percentages, means, medians and standard deviations) for all included variables. The study was not aimed to contrast hypotheses but to inform the development of a PtDA; however, we explored the associations of the three variables derived from the CPS (i.e., preferred role, perceived role and matching between them, each one with three categories) with the remaining variables. Associations with categorical variables (i.e., gender, education, time spent searching GAD-related information—dichotomized into *“once a month or more”* vs. *“less than once a month or never”*—and the three most frequent reasons for searching) were analyzed by means of Chi-square tests. Age and the total number of information themes searched for on the Internet were analyzed with one-way ANOVA. The perceived difficulty of past decisions (in each one of its categories and subcategories) were analyzed by means of the Kruskal–Wallis test (with Mann–Whitney’s U-test in post-hoc comparisons), due to the non-normality of several items and the small sample sizes in several decisions (this analysis was not performed when the number of participants for a certain decision was lower than 30).

## 4. Results

The online survey was opened from April to July 2020. The survey was answered by 77 participants, but only 70 met the inclusion criteria. These seven people were excluded from the analysis because they did not meet the study inclusion criteria (did not present with current or past diagnosis of GAD).

### 4.1. Sample Characteristics

The online survey was successfully completed by 70 respondents from 15 Spanish Regions. Mean age was 41.09 ± 12.21 (median = 40, range 18–76) and 67.1% were female. Most of the respondents had secondary education (38.6%) or university studies (52.9%). Mean time since symptoms onset was 15.59 ± 12.38 years (median = 13.5, range 0.25–50). In the present, 35.7% were receiving combined therapy, 24.3% were receiving only medication, 20% only psychotherapy and 2.9% were being treated with other forms of therapy. Approximately half of the sample (47.1%) never searched for GAD-related information on the Internet or did it less than once a month (Table 1).

### 4.2. Role in Decision-Making Process

Most participants preferred an active (42.9%) or collaborative role (41.4%) in the decision-making process, whereas only 15.7% preferred a passive role. Regarding their perceptions of involvement, 52.9% were active, 22.9% perceived a collaborative role and 24.3% were passive. Matching between perceived and preferred roles occurred in 47.1%, 22.9% perceived less involvement than desired and 30% were more involved than they preferred.

These three variables were not significantly associated with age or gender. Education (dichotomized into secondary education or less versus university studies) significantly related to the perceived role (χ^2^ = 7.75, *p* = 0.021); the group that perceived a passive role included more patients with less than high school education (73.5% vs. 37.5% for shared and 37.8% for active; *p* = 0.021) (Appendix A Table A1).

### 4.3. Treatment-Related Decisions

Table 2 shows the difficulty attributed by participants to the treatment-related decisions they made in the past. The specific decisions about medication and psychotherapy (i.e., start, change, leave) are shown in the appendix (Table A2). A majority of participants had to decide whether to start with psychotherapy (90%) and medication (87.1%) (Appendix A Table A2). The most difficult decision was wait-and-see (M = 2.03, SD = 0.94), and the easiest was choosing the combined pharmacological and psychological treatment (M = 1.29, SD = 1.09), decisions faced by 48% and 54% of the sample, respectively (Table 2).

There was only one significant association between perceived difficulty and decisional roles; patients who perceived a passive role rated the decision of changing medication as easier (1.20) than those who perceive a shared (2.22) or active (2.10) role (*p* = 0.038) (Appendix A Table A1). At the 90% confidence level, the decision of starting psychotherapy was significantly easier for participants who perceived a collaborative role (*p* = 0.064), compared to their counterparts. Patients with more involvement than desired perceived more difficulty in all but one decision, although differences were not significant (Appendix A Table A2).

### 4.4. Frequency of Information Search

Table 3 shows the rate of participants who searched for GAD-related information once a month or more. There were no significant associations with the control role. In the case of the perceived role, active patients showed a higher rate (64.9% vs. 37.5% and 41.2%, *p* = 0.101); when the other two categories were collapsed, the difference reached significance (64.9% vs. 39.4%, *p* = 0.033).

### 4.5. Online GAD Information Needs

Among the reasons for searching for information, 45 participants (64.3%) stated that they were interested in solving doubts about GAD (Table 4). More than a quarter felt that the information provided by their healthcare professional was not enough (28.6%), and 25.7% reported that they needed to find another healthcare professional. None of these variables significantly related to decisional roles (data not shown). Few participants disagreed with the information provided (11.4%) or thought that it was difficult to understand (2.9%). There was a significant association between matching of preferred and perceived roles and interest in learning about GAD (χ^2^ = 6.77, *p* = 0.034): those who perceived less involvement than they desired showed less interest in learning (37.5%), vs. 75.8% in matched participants, and 61.9% in those more involved than desired.

The most relevant GAD-related unmet information needs were: “general information” (71.4%), “information on self-help groups” (65.7%), “recommendations on how to face this disorder” (61.4%) and “information on treatment options” (50.0%) (see Table 5). Association between the total number of themes searched and role matching showed a *p*-value of 0.046; participants who perceived less involvement than desired showed a significantly lower score (2.44) than those more involved than desired (*p* = 0.049); the comparison with matched participants was not significant (3.82, *p* = 0.148).

## 5. Discussion

As a previous step to the development of PtDA, we used a survey in order to identify and characterize the decision-making and unmet information needs of people with GAD, and their Internet use to satisfy them. Results show that most patients prefer an autonomous or collaborative role in the decision-making about treatment, and more than half of the sample did not perceive their desired involvement, by excess or by default. These results are similar to those obtained in previous research, specifically in anxiety [19,21] or other mental disorders [17], and indicate that despite the increased interest and research in patient-centered care and SDM in the last two decades, many mental health patients still feel that their participation preferences are not taken into account. Regarding the sociodemographic correlates of decisional roles, we did not find significant results for role preference, contrary to other studies in mental health [24,25], but found that more educated patients significantly perceived a more active or collaborative role.

Approximately half of the sample searched for GAD-related information on the Internet at least once a month, and one third did it once a week or more: results very similar to those obtained by Liebherz et al. [21] on anxiety disorders. Importantly, among the reasons for seeking information, 29% of participants thought that the information provided by their doctors was insufficient, and 26% were looking for a health professional (23% in Liebherz et al. [21] for both variables). These data reflect a relevant gap of usual services in the provision of information. On the other hand, information obtained from the web may come from non-reputable sources or be inaccurate, and therefore it is important to provide patients with good quality information, in the form of PtDAs or other resources, that could facilitate the work of health care providers without excessive time consumption. The most searched themes were general information, information on self-help groups, recommendations on how to face this disorder and information on treatment options. These results are similar to those found in the systematic review of Tlach et al. [19] on patients with depression or schizophrenia, who reported that the most relevant categories of information needs were basic facts, treatment and coping.

We found few significant associations between decisional roles and the remaining variables, but these analyses were merely exploratory and did not have adequate statistical power. However, some patterns were observed in the descriptive data. Patients who perceived an active participation or more involvement than desired were more likely to search for information frequently and to perceive a greater difficulty in treatment decisions. While in other mental or physical health conditions an excessive involvement from the patient’s perspective could not have unintended consequences, in the case of GAD patients it could represent an excess of responsibility that in turn increases anxiety and worry. This possibility reinforces the need to adequately assess patients’ preferences and perceptions of involvement in the decision making about treatment options. Future research should evaluate the associations of decisional roles and patients’ decisional conflict, treatments chosen and symptoms’ evolution.

To our knowledge, this is the first study reporting on information and decision-making needs conducted particularly with people with GAD. In summary, results support the assumption that people with GAD have several unmet information needs concerning their disorder and treatments, and many of them use the Internet to meet those needs. These data reinforce the idea of developing resources such as PtDAs for people with GAD, to allow them to make informed decisions tailored to their needs. Moreover, research suggests that people with mental disorders want to participate in other aspects of their health care, including research design and evaluation [26]. The SDM process should take place at all levels of care, offering evidence-based information about treatments and discussing the available options.

## 6. Limitations

This study has several limitations. First, the sample size was small and not randomly selected; gender distribution shows the rates observed in epidemiological studies (twofold in women), but most of the sample have secondary or university education, so it might be relevant to analyze the results in a sample with a lower education level. Nonetheless, results follow the line of those found in the literature [19,21,22]. Second, GAD diagnosis was not confirmed, and we cannot assure whether participants presented other anxiety or mental disorders. On the other hand, only the patients’ perspective is included in our study and we have not explored the viewpoint of family members. The statistical contrasts were exploratory and underpowered, and must be interpreted cautiously.

Despite these limitations, our results add evidence to understand the most relevant information needs for people with GAD, a condition understudied with regard to SDM and patient-centered care.

## 7. Conclusions

The results of this study indicate that Spanish people with GAD show an overall preference to participate in a SDM process about treatment, but these preferences are not fulfilled for a considerable percentage of them. Approximately one third of participants regularly use the Internet to access GAD-related information, and more than a quarter felt that their health care providers did not provide them with sufficient information about the disorder and its treatment. These data point out to important gaps in the SDM process carried out in usual services. The study provides a starting point to develop a web-based PtDA to support the SDM process with these patients. Future research should analyze how patients’ preferences and perceptions of involvement, as well as their matching, relate to unmet information needs and the perceived difficulty of treatment decisions.

## Figures and Tables

**Table 1:** Patients’ characteristics (*N* = 70).

**Variables**	
**Age**—mean (SD)	41.09 (12.21)
**Female**—*n* (%)	47 (67.1)
**Education level**—*n* (%)	
Primary studies Secondary studies University studies	6 (8.5) 27 (38.6) 37 (52.9)
**Symptoms’ duration, years**—mean, SD	15.59 (12.38)
**Current treatment**—*n* (%)	
None Psychological Pharmacological Combined Other	12 (17.1) 14 (20) 17 (24.3) 25 (35.7) 2 (2.9)
**Search GAD-related information**	
Never Less than once a month Once a month Once a week 2–3 times a week Daily	7(10) 26 (37.1) 14 (20) 7 (10) 11 (15.7) 5 (7.1)

**Table 2:** Perceived difficulty of past decisions about GAD treatment.

Treatment Decisions (*n*)	Mean Difficulty ^1^	Preferred Role				Perceived Role				Role Matching			
		Active	Shared	Passive	*p*-Value ^2^	Active	Shared	Passive	*p*-Value ^2^	Less than Desired	Matched	More than Desired	*p*-Value ^2^
Wait-and-see (34)	2.03 (0.94)	2 (0.97)	1.70 (1.03)	1.86 (0.69)	0.120	2 (0.91)	1.75 (1.05)	1.50 (0.92)	0.227	1.75 (1.16)	1.69 (0.82)	2.14 (1.03)	0.406
Medication (66) ^3^	1.66 (0.89)	1.58 (0.88)	1.66 (0.99)	1.84 (0.71)	0.802	1.72 (0.91)	1.68 (0.81)	1.50 (0.97)	0.765	1.41 (0.97)	1.58 (0.79)	1.94 (0.96)	0.153
Psychotherapy (64) ^3^	1.34 (0.92)	1.42 (0.86)	1.36 (0.97)	1.12 (0.94)	0.583	1.56 (0.97)	1.07 (0.82)	1.14 (0.81)	0.124	1.37 (0.88)	1.13 (0.86)	1.64 (0.98)	0.157
Combined therapy (38)	1.29 (1.09)	1.58 (1.16)	1.27 (1.08)	0.50 (0.58)	0.230	1.52 (1.21)	1 (0.94)	1(0.82)	0.407	1.22 (0.83)	1.07 (1.03)	1.57 (1.28)	0.519

NOTE: data are means (SD). Items’ source: own contribution; ^1^ Scores range 0 (very easy) to 3 (very difficult); ^2^ *p*-values from Kruskal-Wallis’ test; ^3^ Mean of the averaged scores for each participant in the specific decisions (i.e., start, change, leave) she/he had faced.

**Table 3:** Participants who search for information once a month or more, by decisional role (Control Preference Scale).

**Preferred Role**			
**Active**	**Collaborative**	**Passive**	**χ^2^ (*p*-Value)**
15 (50%)	16 (55.2%)	6 (54.5%)	0.17 (0.917)
**Perceive Role**			
**Active**	**Collaborative**	**Passive**	
24 (64.9%)	6 (37.5%)	7 (41.2%)	4.59 (0.101)
**Role Matching**			
**Less than desired**	**Matched**	**More than desired**	
6 (37.5%)	17 (51.5%)	14 (66.7%)	3.14 (0.207)

**Table 4:** Reasons to search for GAD information.

**Reasons Why You Have Sought Information about GAD (Yes/No) (*n* = 70)**	**nº Yes (%)**
I am interested in learning about GAD	44 (62.9)
I am interested in solving the doubts I have about GAD	45 (64.3)
The information provided by your attending physician/psychologist/psychiatrist was not enough	20 (28.6)
The information provided by your attending physician primary/psychologist/psychiatrist was difficult to understand	2 (2.9)
You disagreed with the information your primary care physician/psychologist/psychiatrist gave you	8 (11.4)
Your primary care physician/psychologist/psychiatrist recommended that you read about GAD	5 (7.1)
I was looking for a health professional (psychologist/psychiatrist)	18 (25.7)
Other *	13 (18.6)

NOTE: Items adapted from Liebherz et al. [20]. * Other reasons included: interest in seeking information about available treatments, side effects of the medication, knowing other points of view or experiences of people with the same problem, opinions of professionals, consultations with the psychologist are very spaced in time. GAD: Generalized Anxiety Disorder.

**Table 5:** Online GAD Information Needs.

Online GAD Information Needs (Yes/No) (*n* = 70)	nº Yes (%)
General information about symptoms, causes, course and progression of GAD	50 (71.4)
Information on how generalized anxiety disorder is diagnosed	17 (24.3)
Information on treatment options (i.e., psychotherapy, medication, other treatments)	35 (50)
Information on benefits, risks and side effects of different treatment options	27 (38.6)
Information on where to obtain treatment (i.e., psychotherapists, physicians, care centers and units, hospitals...)	26 (37.1)
Information about self-help groups/exchange with people who are going through the same thing, experiences of those who have gone through a similar situation	46 (65.7)
Information for family members	6 (8.6)
Recommendations on how to cope with GAD (i.e., coping with concerns, coping strategies for different aspects of daily life, tools to improve self-esteem, etc.)	43 (61.4)
Other *	6 (8.4)

NOTE: Items adapted from Liebherz et al. [20]. * Other themes included: brain areas involved, mechanisms of action of drugs, benzodiazepine addiction, differential diagnosis with other anxiety disorders, influence of family history on anxiety disorders, alternative therapies and natural medicine. GAD: Generalized Anxiety Disorder.

## Appendix A

**Table A1:** Associations between sociodemographic variables and decisional role.

Sociodemographic Variables	Preferred Role				Perceived Role				Role Matching			
	Active	Shared	Passive	*p*-Value ^1^	Active	Shared	Passive	*p*-Value ^1^	Less than Desired	Matched	More than Desired	*p*-Value ^1^
Age, mean (sd)	40.4 (14.5)	41.8 (10.4)	41 (10.7)	0.915	39.8 (11.7)	39.3 (11.6)	45.5 (13.4)	0.235	43.4 (16.3)	41.5 (10.9)	38.6 (10.6)	0.472
Women, *n* (%)	20 (66.7%)	20 (69.0%)	7 (63.6%)	0.947	23 (62.2%)	12 (75.0%)	12 (70.6%)	0.620	12 (75.0%)	19 (57.6%)	16 (76.2%)	0.273
Less than high school, *n* (%)	12 (40.0%)	14 (48.3%)	7 (63.6%)	0.401	14 (37.8%)	6 (37.5%)	13 (76.5%)	0.021	9 (56.3%)	18 (54.5%)	6 (28.6%)	0.125

^1^ *p*-values from ANOVA (age) and Chi-squared test (gender and education).

**Table A2:** Associations between perceived difficulty of past decisions about medication and psychotherapy and decisional role.

Treatment Decisions (n)	Mean Difficulty ^1^	Preferred Role				PERCEIVED ROLE				Role Matching			
		Active	Shared	Passive	*p*-Value ^2^	Active	Shared	Passive	*p*-Value ^2^	Less than Desired	Matched	More than Desired	*p*-Value ^2^
**Medication**													
Start medication (61)	1.89 (1.02)	1.85 (1.01)	1.84 (1.02)	2.10 (1.10)	0.704	1.94 (1.05)	1.85 (0.90)	1.77 (1.09)	0.804	1.58 (1.00)	1.96 (0.96)	1.95 (1.12)	0.458
What medications to take (46)	1.54 (0.98)	1.29 (0.78)	1.67 (1.03)	2.00 (1.29)	0.209	1.39 (0.956)	1.91 (0.94)	1.57 (1.13)	0.334	1.25 (0.71)	1.52 (0.99)	1.73 (1.10)	0.462
Change medication (40)	1.90 (1.03)	1.93 (1.10)	1.94 (1.00)	1.71 (1.11)	0.872	2.10 (1.09)	2.22 (0.83)	1.20 (0.79)	0.038	1.50 (0.93)	1.76 (1.03)	2.27 (1.03)	0.148
Change dose (43)	1.56 (0.96)	1.50 (0.89)	1.60 (0.94)	1.57 (1.27)	0.959	1.65 (0.93)	1.60 (0.97)	1.30 (1.06)	0.695	1.33 (0.87)	1.42 (0.84)	1.87 (1.12)	0.334
Discontinue medication (45)	1.84 (0.95)	2.00 (0.97)	1.70 (1.03)	1.86 (0.69)	0.595	2.00 (0.91)	1.75 (1.05)	1.50 (0.93)	0.367	1.75 (1.16)	1.70 (0.82)	2.14 (1.03)	0.298
**Psychotherapy**													
Start psychotherapy (63)	1.32 (1.01)	1.46 (0.83)	1.21 (1.13)	1.27 (1.10)	0.640	1.59 (1.07)	0.88 (0.81)	1.20 (0.94)	0.064	1.29 (0.99)	1.07 (0.88)	1.70 (1.13)	0.101
Change type of psychotherapy (22)	1.86 (0.94)	n.c.	n.c.	n.c.	n.c.	n.c.	n.c.	n.c.	n.c.	n.c.	n.c.	n.c.	n.c.
Change modality of psychotherapy (22)	1.55 (1.01)	n.c.	n.c.	n.c.	n.c.	n.c.	n.c.	n.c.	n.c.	n.c.	n.c.	n.c.	n.c.
Leave psychotherapy (39)	1.56 (1.07)	1.57 (1.16)	1.68 (0.95)	1.17 (1.33)	0.671	1.75 (1.07)	1.25 (1.03)	1.29 (1.11)	0.350	1.57 (0.98)	1.19 (1.11)	1.94 (1.00)	0.144

n.c.: not calculated because the total sample size is less than 30. ^1^ Score range 0 (very easy) to 3 (very difficult). ^2^ *p*-values from Kruskal-Wallis’ test.
